# District-level implementation of British Columbia’s school food and beverage sales policy: a realist evaluation exploring intervention mechanisms in urban and rural contexts

**DOI:** 10.17269/s41997-018-0159-x

**Published:** 2018-12-07

**Authors:** Adrienne V. Levay, Gwen E. Chapman, Barbara Seed, Hannah Wittman

**Affiliations:** 1 Faculty of Land and Food Systems, University of British Columbia, Vancouver, British Columbia Canada; 2 College of Social and Applied Human Sciences, University of Guelph, Guelph, Ontario N1G 2W1 Canada; 3 B. Seed Consulting, Vancouver, British Columbia Canada; 4 Centre for Sustainable Food Systems, University of British Columbia, Vancouver, British Columbia Canada

**Keywords:** School food policy, School district, Implementation, British Columbia, Urban, Rural, Realist evaluation, Context, Mechanism, Politique alimentaire scolaire, District scolaire, Mise en œuvre, Colombie-Britannique, Milieu urbain, Milieu rural, Évaluation réaliste, Contexte, Mécanisme

## Abstract

**Intervention:**

British Columbia’s (BC) provincial school food and beverage sales policy.

**Research question:**

What are the processes associated with district-level implementation of BC’s school food and beverage sales policy?

**Methods:**

We adopted a realist approach and a qualitative, multiple case study design that included three urban and two rural BC school districts. Data collection involved semi-structured interviews and questionnaires with health, education, and industry stakeholders, observations, document analysis and website scans. Analysis identified: (i) mechanisms influencing if and how stakeholders engage in implementation activities at the district level and (ii) specific dimensions of context influencing these mechanisms.

**Results:**

We identified three mechanisms driving implementation processes at the school district level associated with BC’s school food and beverage sales policy. These mechanisms are influenced by various dimensions of context that lead to a range of implementation outcomes. The ‘mandatory mechanism’ refers to the mandatory nature of the policy effectively triggering implementation efforts, influenced by a normative acceptance of the education system hierarchy. The ‘money mechanism’ refers to how this district demand leads vendors to create a compliant supply; it is influenced by beliefs about children’s food preferences, health and food, and the existence of competition. Finally, the ‘monitoring mechanism’ refers to how systems of informal monitoring are used to promote compliance in the context of a competitive sales environment.

**Conclusion:**

The outcomes of these three policy mechanisms are influenced by complex dimensions of context. Identifying context–mechanism interactions can help inform public health policymakers interested in interventions for improving school food environments.

## Résumé

**Intervention:**

La politique provinciale de vente d’aliments et de boissons dans les écoles en Colombie-Britannique (C-B).

**Question de recherche:**

Quels sont les processus associés à la mise en œuvre, à l’échelle des districts, de la politique sur la vente d’aliments et de boissons dans les écoles en C-B?

**Méthode:**

Nous avons adopté une démarche réaliste et un protocole d’étude qualitative de cas multiples incluant trois districts scolaires en milieu urbain et deux en milieu rural en C-B. Les données ont été recueillies au moyen d’entretiens semi-directifs et de questionnaires (administrés à des acteurs de la santé, de l’éducation et de l’industrie), d’observations, d’analyse de documents et d’examens sommaires de sites Web. Nous avons ainsi repéré : i) les mécanismes qui influencent la participation des acteurs aux activités de mise en œuvre de la politique dans leur district et ii) les aspects particuliers du contexte qui influencent ces mécanismes.

**Résultats:**

Nous avons repéré trois mécanismes déterminants dans les processus de mise en œuvre, à l’échelle du district scolaire, de la politique de vente d’aliments et de boissons dans les écoles en C-B. Ces mécanismes sont influencés par divers aspects contextuels qui entraînent un éventail de résultats de mise en œuvre. Le « mécanisme de l’obligation » désigne le fait que la nature obligatoire de la politique déclenche dans les faits les efforts de mise en œuvre; ce mécanisme est influencé par une acceptation normative de la hiérarchie du système d’éducation. Le « mécanisme de l’argent » désigne le fait que la demande du district pousse les fournisseurs à créer une offre conforme; il est influencé par les convictions au sujet des préférences alimentaires des enfants, du lien entre l’alimentation et la santé et de l’existence de concurrents. Enfin, le « mécanisme du contrôle » désigne l’utilisation de systèmes de contrôle officieux pour favoriser la conformité dans un environnement commercial concurrentiel.

**Conclusion:**

Les résultats de ces trois mécanismes stratégiques sont influencés par des aspects contextuels complexes. Le repérage des interactions entre le contexte et les mécanismes peut éclairer les responsables des politiques de santé publique intéressés par des interventions visant à améliorer l’environnement alimentaire dans les écoles.

## Introduction and background

Interventions for improving school food environments are reported to have numerous benefits to children’s health, including improved eating habits (World Health Organization (WHO) 2008) and decreased body mass indices (Sanchez-Vaznaugh et al. 2010). One increasingly popular approach to improving school food and beverage environments is an implementation of criteria and policy standards aimed at improving the nutritional quality of foods and beverages sold in schools.

Most provincial and territorial governments in Canada have developed and implemented school food and beverage sales guidelines (Holmes 2016). *The Guidelines for Food and Beverage Sales in BC Schools* (the Guidelines) were created in 2005 by the British Columbia (BC) Ministries of Health (BCMoH) and Education (BCMoED), with the most recent iteration launched in January 2014 (Government of British Columbia 2013). These mandatory guidelines apply to all public schools, aiming to reduce the amount of salt, sugar and fat in items sold to students in all food sale venues in schools, including vending machines, cafeterias, fundraisers, sports days and school stores. Previous BC-based studies have found that while school food sale environments have improved since the launch of the Guidelines, improvements have not necessarily occurred in all schools (BCMoED and BCMoH 2008; Watts et al. 2014). As there are 60 school districts with varying geographies and demographics in BC, the BCMoED decentralizes power to the district level, whereby districts determine how to carry out top-down directives, including the implementation of the school food and beverage sales policy. Districts may thus incorporate implementation processes differently, with varying levels of intra- and inter-district compliance. Differential implementation of a public health intervention, like the Guidelines, has the potential to exacerbate health inequities if successfully implemented in some contexts but not others (MacDonald et al. 2016).

Research from other jurisdictions shows the important role school districts play in implementing food and beverages sales policies. In the United States, studies have found district policies influence students’ consumption of sugar-sweetened beverages and significantly reduce the availability of unhealthy items for sale (Larson et al. 2016). Also in the US, researchers have proposed that weak district policies and lack of accompanying resources for enforcement from the district level potentially perpetuate poor school food and beverage environments (Bergman et al. 2015).

In Canada, studies from various provinces have identified district support for implementation as an important facilitator of implementation, including in BC (Masse et al. 2013; McIsaac et al. 2015). However, little research has elaborated on this in the Canadian context and explored social processes associated with implementation occurring at the level of school districts to help explain how and why district practices may contribute to different levels of policy compliance. A nuanced understanding of this can offer leverage points that may be useful for intervening to improve implementation and, consequently, compliance.

### Realist evaluation

A realist approach to evaluating the implementation of complex public health interventions, like BC’s Guidelines, can begin to provide this nuanced understanding. Realist evaluation can explain the inconsistent success of an intervention by assessing the relationships between contexts (C), mechanisms (M) and outcomes (O). First, context is considered the backdrop of an intervention, pre-existing dimensions of which may influence mechanisms and, therefore, outcomes. Although the concept of context is broad and can include, for example, social or cultural norms, histories of organizations or people and governance structures, realist evaluation aims to identify what dimensions of a broad range of potential contextual factors are the most relevant for the success of a specific type of intervention. Second, mechanisms, according to realist methodology, are defined as the ways in which stakeholders respond to the concrete and non-concrete resources provided by an intervention, like funding (a concrete resource) or the fear of ill health created through a health education campaign (a non-concrete resource). Responses to intervention resources can be cognitive, emotional or motivational, or anything else that happens in the mind of stakeholders who encounter them. This can help explain why stakeholders make decisions about if and how they will take action. Additionally, the ways individuals respond to intervention resources are influenced by pre-existing specific dimensions of context (Jagosh et al. 2012). The interaction of contexts and mechanisms leads to outcomes simply defined as effects of an intervention, whether intended, unintended, short-term, intermediate or long-term.

Realist evaluators theorize that interventions will lead to desired outcomes only when intervention resources are launched in facilitative contexts (Pawson and Tilley 1997). A facilitative context is a context that will promote stakeholders to decide to take action to implement (or take up) an intervention. If an intervention resource is offered in a facilitative context, it should trigger the intended response in the minds of stakeholders and they will decide to make an effort to implement. Equipped with a deeper understanding of what is and is not a facilitative context for an intervention, stakeholders can make decisions prior to its launch as to whether it can be introduced ‘as is’ or whether other pre-intervention or coinciding activities are necessary to either foster a facilitative context or adapt the intervention to the existing context (Jagosh 2017).

This paper explores the social processes associated with the district-level implementation of school food and beverage sales standards, focusing on the implementation of the Guidelines for Food and Beverage Sales in BC Schools. Specifically, we explore the influence of contexts on mechanisms that prompt district-level stakeholders to take action to implement. What dimensions of context are facilitative? What perceived intended or unintended outcomes are occurring as a result of the district-level processes of implementation? Are there potential implications for different types of district-level implementation processes to contribute to health inequities?

## Methods

The analyses reported in this paper were part of the first author’s (AL) doctoral research that included the development of a retrospective logic model and program theory for school food and beverage sales interventions as well as an examination of school-level implementation processes (Levay 2018; Levay et al. 2018). This paper focuses only on the processes associated with the district level of implementation. The initial program theory for the BC Guidelines was developed using document analysis, interviews with policy architects and observation at public events and was presented to policymakers and school food practitioners to solicit their feedback. This program theory guided the development of the data collection processes and the lines of inquiry for the analyses in this paper. The processes articulated in the initial program theory, that relate specifically to district-level implementation, were: if the district staff were provided with information about the necessity of improving school food environments and how implementing the policy will fill this need, then they will be motivated to implement the Guidelines. This motivation would be augmented by coupling this information with a top-down directive from the Ministry of Education that districts are mandated to implement these Guidelines. Once motivated to implement, they would access the implementation tools and other resources supplied by the health sector (e.g., dietitian services), assess existing school food environments and, if needed, make changes to what is for sale in schools.

### Data collection strategies

We adopted a multiple case study approach where districts were defined as the case unit (Table 1). To explore how different contexts may influence implementation, districts were purposively selected to include two rural and three urban districts from across BC’s five decentralized regional health authorities (RHAs).

**Table 1:** Characteristics of participating school districts (SD)

	Rural or urban	Student population size 2016/17*	# of schools*
SD 1	Urban	> 40,000	> 60
SD 2	Urban	20,000–40,000	40–60
SD 3	Urban	10,000–20,000	20–40
SD 4	Rural	+ 10,000	10–30
SD 5	Rural	+ 10,000	10–30

*Ranges provided to mask the district identity

We used multiple qualitative data collection strategies. AL conducted semi-structured interviews with 32 purposively selected school district-level staff, public health stakeholders and private food vendors. This provided in-depth information about relationships and interactions among stakeholders, contexts and key activities related to Guidelines implementation. Interviews lasted from 30 to 90 min and were recorded and transcribed.

To obtain school-level perspectives of district-level implementation, 72 structured questionnaires were administered online, via telephone or in person with school-level stakeholders. Four districts allowed us to contact school administrators directly. The remaining district allowed AL to attend a public event where data collection was conducted in person with primarily parent stakeholders. Administrators were also asked if they were willing to forward the online link to the questionnaire to others in their school community. As a result, questionnaires were answered largely by administrators (total of 38) and by a small number of other types of school-level stakeholders, including 22 parents, five school food staff, one teacher and six stakeholders who chose not to identify themselves.

We also conducted website scans of 62 schools from the five districts, all five participating school districts and their associated RHAs to obtain more context around organizational structures and school food. Additionally, we scanned the websites of private vendors who participated in the study to garner more information about their products.

### Analysis

Using Atlas.ti qualitative analysis software (Atlas.ti 2015), interview transcripts and long-answer portions of the questionnaires were coded in configurations of context (C), mechanism (M) and outcomes (O). This technique involved first identifying and labelling an outcome in a portion of text. The text located around the identified outcome was examined closely for the participants’ explanation of how the outcome came about. Once Cs, Ms and Os were identified in a section of the narrative, a linked CMO code (e.g., C24-M10-O23) was created (Jackson and Kolla 2012). The linked CMO codes were categorized based on their outcome and which component of implementation they are related to in the initial program theory (Levay 2018; Levay et al. 2018). We created memos for each district that contained the categorized linked CMO codes. The website scans and questionnaire short answers provided contextual information. A within and cross-case analysis was conducted.

## Results

Three processes emerged as key to shaping district-level efforts to implement the Guidelines: (1) the top-down mandatory directive from the province prompts districts to take action by incorporating adherence to the Guidelines into the request for proposal (RFP) process when seeking bids from private food service providers; (2) this creates an economic incentive for private industry to comply and 3) an informal system of regulation and enforcement helps promote compliance. Within these implementation processes, however, complexity emerges due to the influence of specific dimensions of context; this results in different ways of engaging in implementation activities among expected-implementers at the district level.

### Mandatory mechanism: driver of demand creation

The school food and beverage Guidelines offer both concrete and non-concrete intervention resources. How stakeholders respond to these resources, as shaped by pre-existing contexts, constitute the mechanisms of an intervention. A key intervention resource provided to district-level implementers is the power that accompanies the BCMoED declaring them ‘mandatory’. The BCMoED, in the context of a governmental sector hierarchy, expected that districts would develop strategies to ensure the food they bring into their district is compliant. Across the cases, the top-down directive for ‘mandatory’ compliance has led to the responsibility for implementation becoming part of the job role for specific individuals at the school district level. This was the case in all five districts. District staff did not explicitly report feeling forced or pressured outside of describing the implementation of the Guidelines as a ‘ministry directive’, but one school principal perceived that ‘the government threatens the district guy’ (Administrator, SD 4).

Despite this consistency across districts, the outcomes associated with the ‘mandatory mechanism’ differed in urban and rural contexts. In the three urban districts, vending machine suppliers and private cafeteria catering companies are contracted centrally, a process overseen by the district staff person. In these cases, adherence to the Guidelines is incorporated into the request for proposal (RFP) process. Urban district staff presented their role as relatively straightforward whereby actually implementing the Guidelines falls to vendors who secure district contracts. In the two rural districts, food and beverage sales are not centrally organized and thus the schools themselves are fully responsible for creating their own food and beverage sales environment. This difference in ways of organizing school food and beverage sales between rural and urban districts makes sense as stakeholders from urban areas reported access to options of food and beverage service providers capable of serving many schools across small geographic areas. It may be that there are no vendors who can provide services to all schools in a single district spread across the long distances that characterize the two rural districts. CMO configurations involving the ‘mandatory mechanism’ are summarized in Table 2.

**Table 2:** Mandatory mechanism context-mechanism-outcome configurations

	Context	Mechanism	Outcome
CMO: urban districts response to mandate	The education system is hierarchical. And Urban districts located in densely populated regions with multiple vendor options centrally organize procuring cafeteria and vending services.	Mandatory directive/top-down pressure to implement the Guidelines leads districts to incorporate food procurement work into a staff person’s job role.	Districts demand potential vendors prove they will adhere to the Guidelines when applying for contracts. “…everything needs to fit in the Guidelines and everyone is well versed in that now….companies won’t...get in unless…everything meets the Guidelines…” (district staff, SD 1).
CMO: rural districts response to mandate	The education system is hierarchical. And Rural districts do not centrally organize food procurement and schools are responsible for creating their own food and beverage sales environments.	Mandatory directive/top-down pressure to implement the Guidelines leads districts to incorporate supporting guidelines implementation in schools into a staff person’s job role.	This district staff person works with schools directly by engaging in other supportive/enforcement activities.

### Money mechanism: driver of supply creation

In BC, a small minority of cafeterias are non-profit, run by the school district. However, cafeteria and vending machine services in BC are most often provided by private business and, therefore, private industry stakeholders are the focus of this section. The demand created by urban, centrally organizing school districts was reported by vendors, health sector staff, school stakeholders and district staff to be effective in motivating industry stakeholders to provide offering options that comply with the Guidelines. Within a realist evaluation approach, this economic incentive is considered an ‘intervention resource’ that has been launched in the facilitative context of a profit-motivated, competition-based economy.

Vendors fulfilling district-wide contracts were reported as regularly offering compliant items through one of two different approaches. The first approach was to offer reformulated compliant facsimiles of what might be thought of as conventional school food, such as hamburgers, hot dogs, pizza and French fries. Some district, school and health-sector respondents reported an overall lack of creativity and appeal in the presentation of these types of offerings:

…the food is not great [because] much of it is bland, lacks flavour, can be visually unappealing or soggy (baked fries instead of fried)… it’s just not appealing to many people. (Teacher/hot lunch coordinator, SD 2)

One public health dietitian (RHA B) postulated the reason a vendor would take this approach might be due to pre-existing beliefs about school food. The two large service providers (one cafeteria catering company and one vending machine company), both of which had been in the school-food business for decades, conveyed a set of beliefs around the narrowness of children’s preferences that only included conventional school cafeteria and vending machine fare. These implied beliefs emerged through comments such as, ‘better to sell them a can of Coke at school than have them go across the street to the 7-11 for 2-for-1 energy drinks’ and ‘we have to put something in the machine that will sell’ (vending machine vendor).

The decision to create these often unappealing, but compliant facsimiles may also be influenced by the existence of external vendors in the vicinity of the school (e.g., fast food franchises, convenience stores). These external vendors often offered the ‘real’ versions of conventional school food and are viewed as threats to the school-based vendors’ profits. One large vendor summarized this complexity of deciding what products to sell in schools:

…And revenue loss is not related to products. Revenue loss is related to certain products in certain environments…[For example] let’s use two schools…I’ll call them School A and School B…[in the case of] School A, the nearest store is 15 blocks away. [The vending machines] will survive anything…[in the case of] School B [with options nearby the school]... it needs education towards helping kids make a choice on whether they want to go to the vending machines selling healthy choices or whether they want to walk across the street to the store. (Vending machine vendor)

The second approach vendors may use to comply with the Guidelines was demonstrated by a different large cafeteria service provider that offered diverse and reportedly high quality, compliant options. The staff person at the urban, centrally organized district that had recently contracted this service provider reported conducting a district-wide survey that found students desired high quality, diverse and healthier foods. Their survey findings provided guidance for the RFP process which resulted in this new service provider being awarded the cafeteria contract. The menus from school websites from this particular district confirmed that this new company was offering diverse and interesting lunch options not typically sold in school cafeterias, including curries, vegetarian stir-fry, pastas, frittatas and souvlaki. The interview with the district staff provided ideas about potential facilitative dimensions of context that may have enabled this vendor to take a different direction with school cafeteria food. Along with the district demand for improved food quality and diversity, it might also be important that this vendor had never worked in schools before, but rather had only operated corporate cafeterias. Not only had they not been entrenched in school food for decades but they have been providing food to adults, allowing them to conceptualize school cafeterias in an innovative way.

The CMO configurations involving the ‘money mechanism’ are summarized in Table 3. It is important to reiterate that the money mechanism was not at work at the district level of implementation in the rural districts because they did not procure food centrally.

**Table 3:** Money mechanism context-mechanism-outcome configurations*

	Context	Mechanism	Outcome
CMO: private vendors who ‘reformulate’	Large private vendors want to sustain their business. And Private vendors hold beliefs about student preferences for conventional school food (e.g., chicken nuggets, pizza, etc.). And External vendors exist near the school and are perceived by school-based vendors as competition.	The guidelines are nutrient-based criteria whereby compliance is measured only in terms of salt, sugar and fat. And The demand by the district to comply with the mandatory guidelines while trying to compete with external vendors for students’ business incentivizes vendors to change what they are offering.	Items for sale are compliant, reformulated versions of conventional school food. “I have heard some students say, ‘I don’t buy stuff in the cafeteria because it’s really unhealthy’… and the cafeteria has hamburgers and French fries and I … am assuming, because I know that the vendor at the cafeteria knows about the Guidelines and they are supposed to be baked fries and they are supposed to be hamburgers that meet the guidelines…” (dietitian, RHA B).
CMO: private vendors who ‘innovate’	Large private vendors want to sustain their business. And The district has a desire to change cafeteria food culture. And Private vendors hold set of beliefs about food offerings in cafeterias that challenge conventional ideas of cafeteria food.	The demand of the district to both comply with the mandatory Guidelines and to improve cafeteria food quality incentivizes the private vendor.	Offerings for sale in cafeterias are compliant, diverse and well presented. “…we’re bringing in a new [cafeteria] company. That [decision was] really around food quality. We had previously been with a company for about a 20-year period for our cafeterias... I would not say that we were unhappy with our previous vendor… kids were choosing not to eat that food…it wasn’t super appetizing and it wasn’t well-presented… so when [the two competing companies] came we asked them to bring their food offerings… and [the difference] was glaring in terms of the quality they were offering” (district staff, SD 2).

*The CMOs contained here are relevant only in the urban context as rural school districts do not centrally procure food services

### Monitoring mechanism: a dynamic informal process of enforcement

Urban, centralized districts and rural, decentralized districts both reported engaging in informal processes of monitoring and enforcement but in somewhat different manners. In the rural districts, even though district staff are not involved with organizing district-wide procurement, they engage in informal processes of school-level enforcement. For example, an administrator or teacher may file complaints to the district office about a non-compliant fundraiser that they observed occurring in a different school. The district person reportedly engages in following up with the non-compliant school and exerts pressure to promote compliance. In the context of rural districts, where food services are not procured by the district, it appears that enforcement activities only occur between the district staff and school-level employees. No interaction was reported between any private vendors associated with individual schools and school district staff.

In urban districts, incorporation of compliance into the district-wide RFP process is believed to promote adherence, as presented above. Almost universally in the three urban districts, respondents believed that because compliance is built into the centralized procurement process, vending machines and cafeterias ‘almost always’ or ‘always’ meet the Guidelines. The urban district staff did not report any official assessment of whether the products service providers are actually selling in schools are the compliant items promised in their contract bids. This is not surprising because even though the BCMoED states explicitly that following the Guidelines is indeed ‘mandatory’, they ‘are not supported with a compliance and enforcement program’ (p. 1) (MacDonald et al. 2016). However, these urban districts have developed informal systems of self-regulation once contracts are awarded that include a back-and-forth relationship with the private sector actors and with other school-level stakeholders:

I: So you actually monitor a little bit?

R: Only because I have people tell me all the time.

I: …like self-regulation?

R: [Yes] I don't [want to] be roaming around every cafeteria...not a chance. (District staff, SD 3)

Urban district staff also reported receiving complaints from private sector cafeteria staff, such as when non-compliant fundraising initiatives were occurring elsewhere in the school. District staff reported that they do take action to address these complaints to ensure a fair playing field for food and beverage sales. CMO configurations involving the ‘monitoring mechanism’ are summarized in Table 4.

**Table 4:** Monitoring mechanism context-mechanism-outcome configurations

	Context	Mechanism	Outcome
CMO: monitoring by rural and urban district staff	Interest groups within schools fundraise to provide a well-rounded curricular experience. And Food and beverage sales within schools are in competition with one another.	There are no official enforcement mechanisms in place. If stakeholders selling food in schools perceive non-compliant items are being sold by one another, they are led to reach out to the district and lodge a complaint.	District staff make efforts to address non-compliance within schools: “...right now I have got a note on my desk [from a cafeteria staff] about the [graduation] group selling donuts and coffee [saying] ‘we are supposed to follow the rules, why are not they?’.... because we sell almost no coffee, we are not going to make a big issue [about the coffee]...however, the coffee AND donut fundraiser? I am going to raise the issue” (district staff, SD 1).

## Discussion

**Fig. 1:**
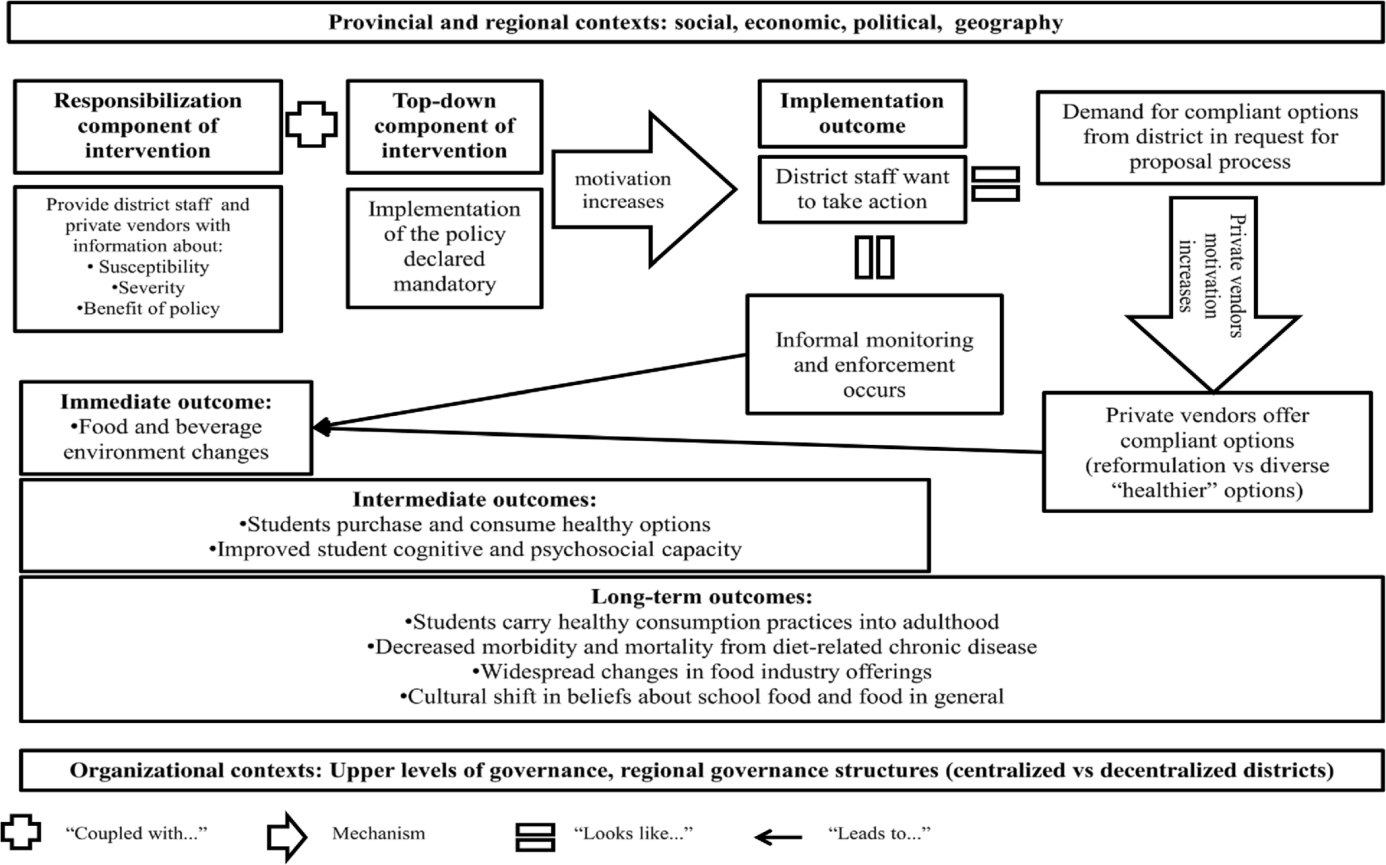
Refined program theory for school food and beverage sales environment interventions at the district level of implementation, adapted from AL’s doctoral dissertation (Levay 2018)

This study aimed to increase the understanding of social processes occurring at the school district level influencing implementation of BC’s school food and beverage sales policy. Specifically, we aimed to elucidate dimensions of contexts that are facilitative (or not) for achieving desired outcomes and what mechanisms drive district-level implementation within these facilitative contexts and develop a refined program theory (Fig. 1).

The acceptance of the hierarchical structure of the education system appears to be a facilitative dimension of context in which to launch mandatory nutrition standards and successfully trigger district-level stakeholders to engage in implementation activities even in the absence of official enforcement measures or sanctions. There is often a low acceptability of paternalistic interventions related to changing the way people eat (Friedman 2014). However, our findings validate Friedman’s hypothesis that, when launched in an acceptable ‘zone of control’ (p. 1743) (Friedman 2014), acceptability and subsequent action come easier (i.e., it is acceptable that there is a high degree of government control over those employed by school districts, civil servants employed to carry out the will of a democratically elected government).

Another facilitative dimension of context is the existence of a market-based economy motivated by profit generation. In urban school districts, this is coupled with an additional facilitative dimension of context, namely that the district centrally organizes cafeteria service providers and vending machines through centralized procurement, creating district-level demand. It is the district demand and the profit motivation that drives private vendors to offer compliant items. Contracting private industry to perform what have typically been public services has been found to be useful, in some cases, for achieving social goals (McCrudden 2004). The literature reports numerous examples illustrating how public procurement impacts private sector operations and improves school food (Morgan and Sonnino 2010). In addition, the expectation of ‘innovation’ in the procurement processes can also be used to foster more effective private sector practices (Edler and Georghiou 2007). The success of contracting private industry to achieve social goals assumes a context of ‘capitalism’. From a realist perspective, in this case a ‘capitalist context’ is a facilitative context for launching school food and beverage sales policies. These findings point to the need to consider the broader political and economic context in which school food and beverage sales policies might be launched and what changes may be required before they can be successful. For example, launching an intervention to improve school food sale environments in contexts that have relatively corrupt or nepotistic contracting practices may result in poorer implementation because economic incentives to comply become less motivating. Another example may be that launching a school food and beverage sales intervention in a context where demand may not be strong enough to incentivize vendors to create a compliant supply, like sparsely populated rural areas, may not be as successful as in more densely populated areas. The processes by which economic incentivization works (or not) to achieve public health goals, in different contexts, is an important area for further research.

Another dimension of context that emerged from this study, the pre-existing beliefs held by private industry stakeholders, is not necessarily ‘facilitative’ but can lead to different ways of implementing nutrition standards. Our findings show that some vendors choose to offer compliant foods and beverages by reformulating conventional school food to meet the salt, sugar and fat requirements of the Guidelines. This reformulation of conventional school food was discussed in another BC-based study (Watts et al. 2014) and in Ontario (Vine et al. 2014). Our study identified potential reasons why vendors make the decision to take this path of implementation, namely persistent beliefs that students will not purchase anything except ‘conventional’ school food offerings and the presence of external vendors. Our data also suggested that reformulated foods may not always be appealing to student customers. This was also found in Ontario, leading students to take their purchasing power off-campus (Vine et al. 2014). In trying to maintain customers, reformulating vendors may ultimately be losing them while contributing to the continued consumption of unhealthy food. On the other hand, a vendor may decide to create reportedly diverse and well-presented offerings that comply with the Guidelines. The findings suggest that this may be a result of the combination of facilitative contexts like the absence of preconceived notions about student food preferences, district-level demand for food and beverage offerings that align with what students want, and a history of working in professional/adult contexts that might have typically expected high-quality offerings. Further exploration into how context influences the cognitive processes of vendors and how they make decisions about their approach to school food service is required for fostering more innovative school food partnerships.

That there is no official or systematic monitoring occurring at the district level is not surprising. Holmes (Holmes 2016) found that it is common in the Canadian context for there to be no systematic monitoring of compliance due to resource constraints and staff not wanting to be ‘the food police’ (p.180). Our findings begin to develop an understanding of how informal monitoring processes have evolved at the district level, in light of a lack of official monitoring, and contributes a more in-depth description of how districts may be attempting to balance compliance and efficiency.

### Theorizing implications of different district-level implementation processes

While the normative hierarchical structure of the education sector allows for effective top-down pressure, districts also have significant freedom to decide how to do their day-to-day business, like creating their food and beverage sales environments. In rural areas where districts are not involved in the creation of the individual school food and beverage environment as a result of being decentralized, there is less control over compliance than in urban districts. Globally, rural-urban health inequities exist where urban populations generally experience better health outcomes (Commission on Social Determinants of Health (CSDH) 2008), including in BC (Chasey et al. 2009). Differential implementation of this public health intervention, where urban school districts may achieve better compliance than rural districts, may exacerbate this inequity.

Another concern with the different ways in which nutrition standards may be implemented at the district level is the more profound messages being sent to students through the type of foods and beverages available at school and the implications for later life. Because BC’s Guidelines are nutrient-based, with the parameters for compliance being focused on three nutrients, the food industry can manipulate the formulas of existing, conventional school food and beverage products to comply. Students provided a ‘reformulated’ food environment are receiving a different set of messaging about healthy food and beverages (Titman 1994), which could put them at a disadvantage as they become adults making food decisions. For example, if they are served nutrient-compliant chicken nuggets and factory-processed pizza every week for 12 years, they may emerge from secondary school with the belief that these are appropriate foods to eat on a regular basis. However, they may not easily be able to distinguish between similar options in the general marketplace, which likely will not adhere to any nutrition standards.

### Study limitations

A possible limitation of this study is the inconsistency of the data sources across cases which occurred due to the variation in how school districts allowed us to collect data. Using a realist perspective to analyze these inconsistent data across cases helps de-problematize this potential limitation because the purpose of realist evaluation is to obtain an insight about broad processes of implementation across contexts. Regardless of whether it was a principal in one district discussing a particular mechanism or a district office staff in a different district discussing the same mechanism, the mechanisms are the same and conclusions can be inferred as to what contextual factors might actually be influencing the underlying mechanisms across context and across individual stakeholders.

### Implications for public health practice

When considering the installation of school food and beverage sales policies, there is a need to consider what type of contexts will be supportive and will trigger the desired mechanisms in expected implementers. In making them mandatory rather than voluntary, policymakers might consider whether the context in which the policy will be launched is one in which official enforcement measures are needed to accompany the mandate to ensure that people take action to implement or if the mandate itself is sufficient to trigger action. Also, if it is expected that the private sector will change their products to comply with the policy because of the demand from school districts, there is a need to consider whether this will be effective in regions where school districts are not procuring private sector food services centrally as a result of a lack of options in rural areas. Moreover, an understanding of how pre-existing beliefs of private sector vendors may be influencing how they choose to comply with policies may help reform the process through which centralized school districts procure private sector services, opting instead for more innovative vendors.
